# Emotion Regulation in the Classroom: A Network Approach to Model Relations among Emotion Regulation Difficulties, Engagement to Learn, and Relationships with Peers and Teachers

**DOI:** 10.1007/s10964-022-01678-2

**Published:** 2022-09-30

**Authors:** Debbie De Neve, Michael V. Bronstein, An Leroy, Alex Truyts, Jonas Everaert

**Affiliations:** 1 Centre of Expertise in Urban Education, Karel de Grote University of Applied Sciences and Arts, Antwerp, Belgium; 2 Department of Psychiatry and Behavioral Sciences, University of Minnesota, Minneapolis, MN USA; 3 Department of Medical and Clinical Psychology, Tilburg University, Tilburg, Netherlands; 4 Research Group of Quantitative Psychology and Individual Differences, KU Leuven, Leuven, Belgium

**Keywords:** Emotion regulation, Student engagement, Teacher support, Peer relations, Adolescence

## Abstract

Emotion regulation is theorized to shape students’ engagement in learning activities, but the specific pathways via which this occurs remain unclear. This study examined how emotion regulation mechanisms are related to behavioral and emotional engagement as well as relations with peers and teachers. The sample included 136 secondary school students (59,7% girls; M_age_ = 14.93, SD_age_ = 1.02, range: 13–18 years). Psychometric network models revealed that difficulties in emotional awareness, emotional clarity, and access to emotion regulation strategies were differentially related to behavioral and emotional engagement, establishing an indirect link with teacher and/or peer relations. Nonacceptance of emotional responses, emotional awareness, and impulse control difficulties were uniquely related to teacher and/or peer relations, establishing an indirect link with student engagement. Causal discovery analysis suggested that student emotional engagement is an empirically-plausible direct cause of increased access to emotion regulation strategies. These findings uncover potential pathways through which emotion regulation hampers or facilitates learning at school, providing information useful for the design of school curricula and teacher training programs.

## Introduction

Adolescence is a critical period for learning effective emotion regulation. The significant emotional, social, and cognitive challenges (e.g., new academic pressures, the need to form new peer and romantic relationships; Rapee et al., 2019) experienced by adolescents requires them to learn to cope with stress and regulate negative emotions (Riediger & Klipker, 2014)—influencing their intensity, duration, or frequency (Gross, 2014). However, younger adolescents spend less effort searching for ways to reduce stressors (Zimmer-Gembeck & Skinner, 2010), make use of more maladaptive regulation strategies, and employ adaptive emotion regulation with limited efficacy (Cracco et al., 2017; Zimmer-Gembeck & Skinner, 2010; Zimmermann & Iwanski, 2014). This habitual pattern of emotion regulation may be problematic because difficulties in emotion regulation have been linked to maladaptive outcomes, including slower stress recovery and increased risk of experiencing common forms of psychopathology (Compas et al., 2017; Gratz et al., 2015), which dramatically increase in prevalence during adolescence (World Health Organization, 2005). Studying key sites where adolescents learn to regulate their emotions, and are impacted by adaptive and maladaptive emotion regulation, is therefore a priority. To date, relatively little is known about how adolescents’ emotion regulation difficulties shape their social and emotional functioning within one of the most important contexts—the school environment. This study addressed this limitation by mapping how emotion regulation mechanisms are related to behavioral and emotional engagement in learning activities as well as relations with peers and teachers.

School activities evoke positive emotions—such as enjoyment when learning new knowledge and skills or pride after succeeding at a test—but also negative ones—like frustration when attempting a difficult test problem, or anxiety about an upcoming test (Boekaerts & Pekrun, 2016). Extracurricular activities may also evoke emotions that emerge within the school environment, including the sadness of a romantic relationship break-up, anger after being slighted by a friend, or guilt about an earlier fight with parents. Learning and performing at school may both influence and be influenced by these emotional experiences. For example, positive emotions about school activities may help students imagine themselves realizing goals, creatively solving problems, and growing as a person. In contrast, negative emotions about studying and taking exams may hinder academic performance and increase school dropout (Clore & Huntsinger, 2009; Zeidner, 2014).

In studying emotion regulation within the classroom, it is important to distinguish different facets of emotion regulation. Emotion regulation involves various strategies that can be distinguished based on the stage of the emotion generation process they aim to modulate (Gross, 2014; 2015). According to the process model of emotion regulation (Gross, 2015), emotion regulation may influence emotion by changing which situations are encountered or how they unfold (situational strategies) or changing which aspects of the situation are attended to by reallocating attention resources between its emotionally relevant and irrelevant facets (attentional strategies). Emotion regulation strategies may also change how a situation is appraised, how it is construed or which goals it is compared to (cognitive strategies) or may change a situation’s experiential, physiological, or behavioral components (response modulation strategies). Other theoretical models (e.g., the adaptive coping with emotions model; Berking & Whitley, 2014) have proposed a set of interacting general skills that are necessary for effective regulation of various emotions. These skills include the ability to be consciously aware of one’s emotions and to correctly identify and label them. In addition, the ability to identify causes of one’s emotional experiences, actively modify emotions in an adaptive manner, and accept/tolerate negative emotions when necessary are considered important. Finally, the ability to approach and confront situations that trigger undesired emotions, as well as the ability to self-sooth in distressing situations, are considered key skills for effective emotion regulation.

Difficulties in emotion regulation may occur at various stages of the regulatory process. Guided by conceptual and empirical work, several dimensions of difficulties in emotion regulation have been distinguished (Gratz & Roemer, 2004). These dimensions provide a means to parse potential difficulties that students may experience when regulating various emotions evoked by school-related activities. Students may lack awareness of or attention to their own emotional responses (lack of emotional awareness) or have difficulties understanding or knowing their emotions (lack of emotional clarity). Moreover, students may have a tendency for negative or non-accepting reactions to their own distress (difficulties accepting emotional responses) or have difficulties controlling their behavior when they are upset (difficulties controlling impulses). Finally, students may hold certain beliefs that could disrupt emotion regulation. For example, they may believe there is little they can do to regulate or control their emotions when they are upset (limited access to emotion regulation strategies). Research has shown that these different aspects of emotion regulation difficulties are related to externalizing and internalizing problems (Neumann et al., 2010).

Emotion regulation difficulties may interfere with both student engagement and social relations with peers and teachers. Student engagement refers to the students’ willingness to be involved in schooling, including by relating to others at school, engaging in learning activities, acting according to institutional values, and working toward academic goals (Skinner et al., 2009). Engagement is an important predictor of student performance (e.g., Klem & Connell, 2004) and mediates the relation between students’ emotions and their academic achievement (Linnenbrink-Garcia & Patall, 2015; Pekrun & Linnenbrink-Garcia, 2012). Student engagement encompasses behavioral and emotional components (Appleton et al., 2008; Fredricks et al., 2004). Behavioral engagement refers to students’ effort, attention, and persistence while initiating and participating in learning activities (Skinner et al., 2009). Emotional engagement includes positive and negative emotions about motivated participation during learning activities in the classroom (Skinner et al., 2009). Distinguishing these two components of student engagement seems important to understand the role of students’ emotion (regulation) within the classroom. For example, research has shown that positive emotions—such as enjoyment of learning—are positively associated with behavioral engagement (e.g., Efklides & Petkaki, 2005; Pekrun et al., 2002a), whereas negative emotions—such as hopelessness and boredom—are negatively associated with behavioral engagement (e.g., Linnenbrink, 2007; Pekrun et al., 2002b). Yet, negative emotions may also have positive effects as they motivate students to perform better (e.g., feeling ashamed about a poor test result may motivate someone to study more; Linnenbrink, 2007; Pekrun et al., 2002b; Turner & Schallert, 2001). Furthermore, motivational resilience models suggest that highly engaged students are not only less affected by stressful events related to school activities (e.g., challenging tasks), but they also use more effective coping strategies, such as help-seeking. The use of these effective coping strategies leads to increased persistence and re-engagement with difficult academic material. In contrast, less engaged students tend to be more discouraged when facing problems and apply more maladaptive coping strategies, such as blaming others, which in turn results in high levels of disengagement (Skinner et al., 2014). Although some studies have focused on the relationship between emotions and student engagement, little is known about how difficulties in various facets of emotion regulation are related to student engagement in the school context.

Just as emotional experiences can influence student engagement, they may influence, and be influenced by social relations with peers and teachers. Emotion regulation has been linked to a sense of school connectedness, which refers to feelings of being accepted, respected, included, and supported by others in the school (Kopelman-Rubin et al., 2020; Zhao & Zhao, 2015). Research has also shown that students with a high sense of connectedness at the start of a school year were more engaged, displayed fewer unpleasant emotions, and received more support from teachers and peers (Furrer & Skinner, 2003). Furthermore, it has been suggested that students’ emotion regulation choices can affect how they are perceived by others (Jacobs & Gross, 2014). For instance, it seems more likely that someone who is perceived as a happy student will receive more support from teachers and be seen as someone peers want to be friends with. By contrast, teachers may have difficulty forming good relationships with children or young adolescents who have behavioral problems (Jerome et al., 2009). It has been suggested that when teachers invest in warm and close relationships with their students, this can help students’ engagement, and increase the use of more effective coping and emotion regulation (Shields et al., 2001; Skinner et al., 2014). These studies suggest that affective processes may modulate students’ social relations at school, though they lack specificity regarding which emotion regulation mechanisms may contribute to lower connectedness with teachers and peers at school.

## Current Study

Although prior research has focused on the relationship between social relations and student engagement and the relationship between emotions and student engagement, much remains to be discovered about the complex interplay between emotions, student engagement, and peer relations. This study was designed to address this critical gap in the literature by uncovering which emotion regulation processes are relevant to understanding aspects of student engagement and social relations within the classroom. Specifically, it examined whether emotional awareness, lack of emotional clarity, difficulties accepting emotional responses, difficulties controlling impulses, and limited access to emotion regulation strategies are (differentially) related to lower levels of behavioral and emotional aspects of student engagement as well as weaker supportive relations with teachers and connections with peers in the classroom. Because student engagement and peer/teacher relations are not independent classroom processes (Juvonen et al., 2012; Wentzel, 2009), this study also considered potential relations between facets of student engagement and social relations in the classroom to uncover their direct and indirect connections with emotion regulation difficulties. To this end, this study applied advanced statistical modeling approaches to identify complex relations between emotion regulation mechanisms, teacher and peer relationships, and student engagement. This comprehensive data-driven approach enables novel insights into how emotion regulation facets connect to socio-affective classroom processes. Insights generated by this approach may further inform policy recommendations and school-based interventions targeting emotion regulation to facilitate student engagement and social connectedness in the classroom.

## Methods

### Participants and Setting

Data were collected in 10 classrooms of 6 Flemish secondary schools (in Belgium). Applying the Degree of Urbanization method (Dijkstra et al., 2021), three schools were located in an urban environment and three schools were located in semi-dense areas. Schools also varied with respect to the number of Flemish educational indicators (see Table 1). In Flanders, the government allocates additional teacher-hours to secondary education schools so that they can develop a policy for equal educational opportunities. This is based on four indicators (AGODI, 2021), namely home language (whether the language spoken by the members of a family at home is different from the language of instruction), mother’s level of education (whether the mother holds a diploma or certificate of higher secondary education), neighborhood (whether students live in a neighborhood with many school delays), and school allowance (whether students from low-income families receive an additional scholarship).

**Table 1:** Participants and education indicators per school

	School 1	School 2	School 3	School 4	School 5	School 6	Average per indicator
Number of participants	23	19	18	20	43	13	
Indicator home language	10%	14%	8%	57%	68%	43%	11%
Indicator mother’s level of education	15%	5%	18%	75%	71%	60%	24%
Indicator neighborhood	14%	30%	14%	91%	89%	87%	22%
Indicator school allowance	25%	11%	26%	80%	71%	67%	31%

*Notes:* school 3: number of participants of 2 classes; school 5: number of participants of 4 classes; average per indicator for school year 2019–2020 were retrieved from AGODI (2021)

Teachers from the participating schools recruited students from grades 9 and 10. The students and their parents received an information letter and consent form. 136 of 153 parents (88.9%) and all 136 students provided consent to participate. The sample (Age: *M* = 14.93, *SD* = 1.02, range: 13–18 years) included 59.7% female and 38.1% male respondents. A small number (2.2%) of the participants preferred not to mention or did not report their gender. The participants answered all items of the measures below (i.e., there was no missing data).

### Measures

#### Emotion regulation

Emotion regulation was measured using the subscales of the Difficulties in Emotion Regulation Scale Short Form (Kaufman et al., 2016). This questionnaire was developed to comprehensively measure various facets of emotion regulation difficulties during distress. This study included the subscales focusing on awareness of emotions (e.g., “I pay attention to how I feel”), lack of emotional clarity (e.g., “I am confused about how I feel”), nonacceptance of emotional responses (e.g., “When I’m upset, I feel guilty for feeling that way”), impulse control difficulties (e.g., “When I’m upset, I become out of control”), and limited access to strategies to regulate emotional responses (e.g., “When I’m upset, it takes me a long time to feel better”). These subscales measure emotion regulation difficulties that occur in response to various (negative) emotional experiences. Each subscale consisted of three items to be rated on a five-point Likert scale. Participants indicate how often the items apply to themselves on a scale from 1 (never) to 5 (always). Research examining the properties of the short form of the Difficulties in Emotion Regulation Scale has found that the questionnaire has adequate reliability and validity in samples of community adolescents (Hallion et al., 2018). McDonald’s omega was 0.71 for emotional awareness, 0.77 for emotional clarity, 0.77 for nonacceptance of emotional responses, 0.89 for impulse control difficulties, and 0.76 for limited access to strategies to regulate emotional responses.

#### Supportive relationship with teachers

The supportive environment scale of the Adolescent Resilience Questionnaire (Gartland et al., 2011) was used to measure supportive relationships with teachers within the school. The original subscale consists of 8 items that are rated on a five-point Likert scale ranging from 1 (“never”) to 5 (“always”). Example items are: “My teachers are caring and supportive of me” and “My teachers notice when I am doing a good job and let me know about it”. Participants are instructed to rate each item with respect to their own school. One item of the original subscale, namely “At school students help to decide and plan things like school activities and events”, was excluded from this study based on the pattern of loadings resulting from the factor analysis reported in the original study (Gartland et al., 2011). Psychometric research evaluating the ARQ subscales has demonstrated that the supportive environment subscale has a good internal consistency with a Cronbach’s alpha of 0.80 in a sample of adolescents (Gartland et al., 2011). The reliability of the supportive environment subscale of the ARQ in this study was (McDonald’s omega was 0.79).

#### Peer relations in the classroom

Peer relations in the classroom were measured using a single-item drawn from prior work (Mikami et al., 2005). Participants were asked “How many students in this class do you get along with?” and selected the most appropriate option from the following response options: “I get along with everybody in this class” (1), “I get along with most of them” (2), “I get along with half of them” (3), “I get along with few of them” (4), and “I get along with nobody in this class” (5).

#### Behavioral and emotional engagement

Student engagement was measured using the behavioral and emotional engagement scales from prior research (Skinner et al., 2009). The original behavioral and emotional engagement scales (Skinner et al., 2009) each consist of 5 items to be rated on a five-point Likert scale ranging from 1 (not true) to 5 (true). This study utilized the Dutch version of the scales (Denies et al., 2017) which includes the 5-item behavioral engagement scale (example item: “I pay attention in class”) and a 4-item emotional engagement scale (example item: “When we work on something in class, I feel interested”). All items are rated on a five-point Likert scale ranging from 1 (not true) to 5 (true). McDonald’s omega was 0.76 for the behavioral engagement scale and 0.66 for the emotional engagement scale.

### Study Context and Procedure

Written informed consent was obtained from the participant’s parents or legal guardians. All children provided assent. Participants completed the questionnaires at school during class hours. The questionnaires were presented in randomized order to participants. Participants were debriefed after completing the study.

Of note, the study was conducted during the COVID-19 pandemic (September–November 2020) when face-to-face education was allowed. During this period, the Belgian government issued various regulations within the school context to stop the spread of the coronavirus. Social distancing and quarantine measures interfered with daily school activities, such that many adolescents were not able to attend school or followed classes online. This context increases the importance of this study on emotion regulation within the classroom because quarantine and online learning may impose challenges to emotional and social functioning (Magson et al., 2020).

### Data-Analysis

The data-analytic plan consisted of fitting a psychometric network model to map unique relations among emotion regulation mechanisms, teacher and peer relations, and both emotional and behavioral student engagement. Follow-up exploratory causal discovery analyses were conducted to probe potential causal pathways via which emotion regulation mechanisms might influence teacher and peer relations and, ultimately, impact aspects of student engagement.

#### Psychometric network analysis

Psychometric networks are abstract models consisting of a set of nodes that represent the study variables and a set of edges that represent statistical relationships between nodes (Borsboom & Cramer, 2013). Network analysis enables the study of relations between a larger number of study variables, allowing inferences about their unique direct and indirect relations accounting for the influence of the other study variables that would not be possible when looking at all variables separately (Haslbeck & Waldorp, 2018). Network analysis represents a more parsimonious approach than fitting a series of regression or mediation models with subsets of study variables. Following variables were included in the network: teacher support, peer relations in the classroom, awareness of emotions, lack of emotional clarity, nonacceptance of emotional responses, impulse control difficulties, limited access to emotion regulation strategies, behavioral engagement, and emotional engagement.

The network was estimated in R (version 4.0.3; R Core Team, 2018) using the estimateNetwork function from the *bootnet* package (Epskamp et al., 2018). The network was fitted as a Gaussian Graphical Model (GGM). In a GGM, the edges represent pairwise relations between two nodes controlling for the other nodes in the network. The GGM was estimated based on correlations coefficients after applying nonparanormal transformation to the data via the huge.npn function from the huge package for R (Zhao et al., 2012). The GGM was regularized using the graphical least absolute shrinkage and selection operator algorithm and coupled with extended Bayesian information criterion model selection (EBICglasso). This procedure shrinks all edges and sets small edges to zero to return parsimonious networks (Friedman, Hastie, & Tibshirani, 2008). This powerful method avoids estimating false positive edges and provides insight into strong relations in the dataset (Epskamp et al., 2016). The GGM tuning parameter was set to the conservative value of 0.5 to increase the specificity of the estimated networks (Epskamp & Fried, 2018). This method enables examination of unique relations between the study variables. In the visualized networks, blue edges represent positive relations and orange edges represent negative relations between the network nodes. Thicker edges indicate stronger associations between the nodes.

The relative importance of the nodes within the network (i.e., emotion regulation facets, teacher and peer relations, and aspects of student engagement) was examined using the (one-step) expected influence metric (Robinaugh et al., 2016) using the R package *networktools* (Jones, 2017). This metric is more appropriate than other centrality metrics (e.g., strength centrality) when networks contain both positive and negative edges (Robinaugh et al., 2016). Expected influence is defined as the sum of all edges extending from a given node (maintaining the sign of each edge). Higher expected influence values indicate greater importance in the network. Importantly, the expected influence values and standard deviations (*SD*s) of the individual nodes in the estimated network were not significantly correlated in the present study (*ρ* = −0.083, *p* = 0.800). This suggests that differential variances of emotion regulation facets, teacher and peer relations, or aspects of student engagement did not affect their centrality in the estimated network.

The accuracy and stability of the network estimates was examined through network stability analyses using the *bootnet* R package (Epskamp et al., 2018). The stability of the edge weights was examined by constructing a 95% confidence interval (CI) around each edge using non-parametric bootstrapping with 1000 samples and by computing bootstrapped difference tests for edge weights. Furthermore, the stability of the centrality metrics was examined using case-dropping subset bootstrapping with 1000 samples and by computing bootstrapped difference tests for expected influence and bridge expected influence values. This method draws samples from subsets from the original data and re-estimates the centrality metric for each subset. The correlation stability (CS) coefficient was calculated to quantify the stability of the expected influence metric. The CS coefficient of expected influence was 0.284 for the estimated network, which is above the recommended threshold (0.25) for stable estimation (Epskamp et al., 2018). The results of the stability analyses are provided in Supplementary 1.

#### Exploratory causal discovery analysis

Causal discovery analyses are an emerging class of machine-learning algorithms that leverage patterns (e.g., of partial correlation) in observational datasets to identify empirically plausible causal relations between their constituent variables (see Fig. 1 in Supplementary 2). These analyses can successfully recover even complex causal pathways, such as those involved in the pathophysiology of Alzheimer’s disease (Shen et al., 2020), from observational data.

This study used the Greedy Fast Causal Inference (GFCI) algorithm to infer empirically plausible causal relations between markers of emotion regulation, behavioral/emotional engagement, as well as peer and teacher relations. The GFCI algorithm searches the space of penalized likelihood scores of all possible acyclic causal relations among the measured variables to produce a preliminary assessment of likely causal pathways. This preliminary result is then iteratively refined by ruling out causal models that imply patterns of conditional independence inconsistent with the data. The output of this procedure is a partial ancestral graph (PAG), with the edge type (Table 1 in Supplementary 2) varying depending on the set of directed edges that were present across all remaining plausible causal models (e.g., a directed edge [arrow] is present if, and only if, all models not containing that edge were removed during the steps outlined above). A particular strength of the GFCI model is its ability to identify situations where unmeasured variables confound the relation between two measured variables, making it particularly well-suited to analyses of data from human research studies (where practical concerns, such as time limitations, constrain measurement of all relevant variables).

To better ensure graph stability, the GFCI algorithm was repeated on 10,000 jackknifed re-samples of the study data. These re-samples were created by randomly deleting 10% of cases from the study dataset. The original dataset was included as an additional re-sample. Results were aggregated into a single, consensus PAG by depicting the edge type (including: “no edge”) and orientation most commonly present in the PAGs created from the jackknifed re-samples. The full FCI rule set was employed. Default values for remaining parameters were used. For example, the penalty discount (c) used for generating the initial likelihood scores (BIC) was set to 1, the alpha value used in conjunction with Fisher’s z tests to determine conditional independence and refine the preliminary results was set to 0.010, and one-edge faithfulness was not assumed. Because causal discovery algorithms recover causal pathways more effectively when they are provided with prior knowledge (Shen et al., 2020), teacher support was required to be a direct cause of emotional engagement (Tao et al., 2022; Quin, 2017; Strati et al., 2017). To provide information about the size of potential causal effects identified by GFCI, structural equation models featuring the edges GFCI suggested were fit to the data (using lavaan; Rosseel, 2019). Standardized structure coefficients were then added to the PAG.

## Results

### Descriptive Statistics and Zero-Order Correlations Between Study Variables

Means and standard deviations for each study variable are provided in Table 2. This table shows that there was sufficient variability in the item scores of all emotion regulation facets, peer and teacher relations, and aspects of student engagement. As further shown in Table 2, the pattern of zero-order correlations indicates that aspects of student engagement and facets of emotion regulation difficulties were anti-correlated. Moreover, teacher support was negatively correlated with emotion regulation difficulties and positively correlated with student engagement. Peer relations in the classroom was positively correlated with emotional engagement and negatively correlated with nonacceptance of emotional responses.

**Table 2:** Descriptive statistics and correlations between study variables

	M	SD	1	2	3	4	5	6	7	8	9
1 Teacher support	3.70	0.70	1	0.01	0.31**	−0.25**	−0.19*	−0.25**	−0.24**	0.32**	0.34**
2. Peer relations	4.01	1.06		1	0.06	−0.09	−0.24**	0.08	−0.03	0.07	0.30**
3. Emotional Awareness	3.51	0.82			1	−0.38**	−0.18*	−0.09	−0.23**	0.32**	0.31**
4. Lack of emotional clarity	2.53	0.98				1	0.43**	0.29**	0.42**	−0.17*	−0.26**
5. Nonacceptance of emotional responses	2.62	0.99					1	0.38**	0.50**	−0.09	−0.25**
6. Impulse control difficulties	2.43	1.15						1	0.50**	−0.04	−0.21*
7. Limited access to emotion regulation strategies	2.75	0.98							1	−0.13	−0.34**
8. Behavioral engagement	3.99	0.63								1	0.55**
9. Emotional engagement	3.71	0.73									1

*Note.* *p < 0.05; **p < 0.01

### Psychometric Network Analysis

**Fig. 1:**
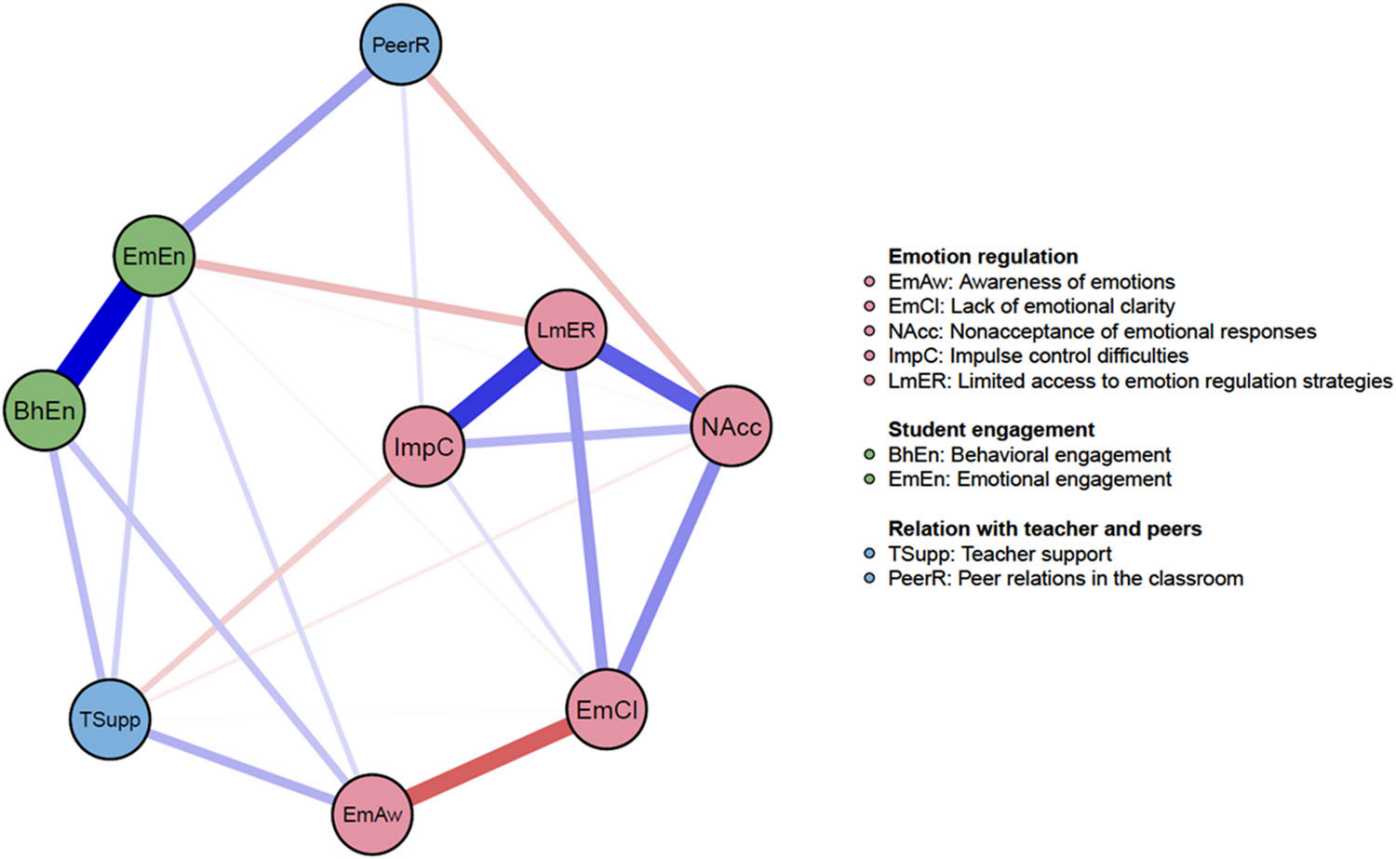
EBICglasso network structure

Figure 1 depicts the EBICglasso network structure. Various edges between facets of emotion regulation difficulties, peer relations, teacher support, and aspects of student engagement survived the conservative regularization procedure. The network plot shows that nodes representing emotional and behavioral student engagement were strongly related. Likewise, the facets of emotion regulation difficulties were strongly interconnected.

Interestingly, emotion regulation facets were directly related to aspects of student engagement as well as indirectly via peer and teacher relationships. Regarding the direct paths, the network shows that emotional engagement was negatively related with limited access to emotion regulation strategies and lack of emotional clarity. Emotional engagement was positively associated with emotional awareness. Behavioral engagement was (positively) related to only one emotion regulation facet, namely emotional awareness. Neither emotional nor behavioral engagement was directly related to non-acceptance of emotional responses or impulse control difficulties.

Regarding the indirect paths in the network, the network plot in Fig. 1 reveals that teacher and peer relations were connected to various aspects of emotion regulation difficulties and student engagement. Nonacceptance of emotional responses was negatively associated with both teacher support and peer relations, which were in turn positively related to aspects of student engagement. Furthermore, impulse control difficulties were connected to emotional and behavioral aspects of student engagement through its negative relation with teacher support. Interestingly, impulse control difficulties were positively associated with peer relations in the classroom, which was in turn positively related to emotional engagement. Finally, in addition to its direct relations with behavioral and emotional engagement, emotional awareness was also indirectly connected to both aspects of student engagement via its positive relation with support from teachers.

With respect to the relation between student engagement and peer/teacher relationships, the network plot shows that teacher support is positively related to both emotional and behavioral components of student engagement. Peer relations were only linked with emotional engagement.

**Fig. 2:**
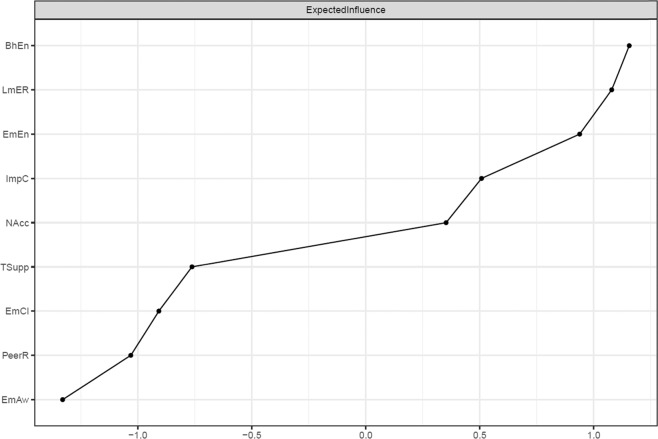
Centrality plot of the fitted network

Expected influence values were computed for all nodes in the network to examine their centrality or relative importance within the network. Fig. 2 depicts the centrality plot. The plot shows that limited access to emotion regulation strategies and both aspects of student engagement were among the most important nodes in the network. This was evidenced by their various and relatively stronger edges connecting to other nodes in the network. The centrality difference test (see Fig. 3 in the Supplementary Information) supports this conclusion and suggests that the expected influence values for the limited access to emotion regulation strategies and student engagement aspects were significantly greater than the centrality values for most other variables in the network.

### Exploratory Causal Discovery Analysis

**Fig. 3:**
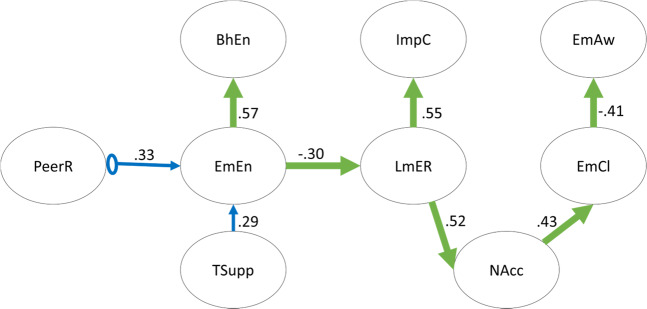
Directed Acyclic Graph suggested by the Greedy Fast Causal Inference (GFCI) causal discovery algorithm. *Notes*. See Table 1 in Supplementary 2 for a description of possible edge types. Numbers adjacent to edges are standardized parameter estimates from a structural equation model of the causal structure suggested by GFCI. PeerR Peer relations. EmEn Emotional Engagement. BhEn Behavioral Engagement. TSupp Teacher Support. LmER Limited Access to Emotion Regulation Strategies. ImpC Impulse Control Difficulties. NAcc Non-acceptance of emotional responses. EmCl Lack of Emotional Clarity. EmAw Emotional Awareness

The PAG generated by GFCI included several potential causal relations between markers of emotion regulation, behavioral/emotional engagement, and peer and teacher relations. The final graph, which is depicted in Fig. 3, suggested that student emotional engagement is an empirically-plausible potential direct cause of increased access to emotion regulation strategies. Through this putative effect, the PAG suggested that emotional engagement may indirectly improve impulse control, acceptance of emotions, and emotional clarity.

## Discussion

Emotion regulation is theorized to shape students’ engagement in learning activities, but the specific pathways via which this occurs remain unclear. This study examined how emotion regulation mechanisms are related to behavioral and emotional engagement as well as relations with peers and teachers during the global COVID-19 pandemic. When direct relations between emotion regulation difficulties and student engagement were inspected, this study observed that emotion regulation difficulties were differentially linked to components of student engagement. Higher levels of emotional engagement were uniquely related to greater recognition of and attention to one’s own emotions (emotional awareness) as well as a better ability to understand one’s own emotions (emotional clarity). Emotional engagement was also related to less endorsement of the belief that one can do little to regulate emotional distress (limited access to emotion regulation strategies). This suggests that those students who display negative emotions about participating during learning activities in the classroom may experience difficulties with emotional awareness, emotional clarity, and access to strategies to regulate emotions. With respect to behavioral engagement, only emotional awareness explained a significant portion of the variance. More active student participation in learning activities was related to higher levels of students’ recognition and attention to their own emotions. This indicates that adolescent students who are more aware of their emotional experiences try harder and are more persistent during learning activities. Of note, other aspects of emotion regulation difficulties, such as non-acceptance of emotional responses and impulse control difficulties, were not related to components of student engagement.

Causal discovery analysis suggested that student emotional engagement may increase access to emotion regulation strategies, with beneficial downstream effects on non-acceptance of emotions, emotional clarity, and impulse control difficulties. This suggestion is consistent with motivational resilience models proposing that highly engaged students tend to use more effective coping strategies, whereas less engaged students employ more maladaptive coping strategies (Skinner et al., 2014). However, the suggestion that awareness and clarity of emotions may be *consequences*, rather than *causes*, of emotion acceptance and access to strategies that are perceived as effective is remarkable. Awareness and clarity of emotions are often considered key steps *preceding* the implementation of effective emotion regulation strategies (Berking et al., 2008). It is plausible that there may be a bidirectional relation. Adolescents who accept their emotions may be more able to accurately characterize them and notice their effects. This may lead them to a greater sense of clarity and awareness as they learn more about their own emotional experiences. Greater clarity and awareness of emotions, in turn, could prompt greater acceptance. However, these results from the causal discovery analyses should be interpreted carefully because the sample was relatively small for analysis with GFCI. The partial ancestral graph may therefore have omitted smaller causal effects (power analyses for GFCI are still under development). Moreover, the patterns of causation identified in this cross-sectional dataset may differ from those that unfold over time. Further research should re-examine this pattern of relations in longitudinal datasets. It should also be noted that GFCI assumes that causal graphs are acyclic (i.e., that there are no vicious/virtuous cycles of causation). Thus, potential causal cycles are not represented in the graph. This may explain why, for example, there was no evidence that emotion regulation feeds back onto teacher support or student engagement.

In the network analysis, emotion regulation difficulties were also linked to relationships with peers and teachers. Nonacceptance of emotional responses was negatively related to both teacher support and peer relationships. Adolescents who have a tendency for negative or non-accepting reactions to one’s own distress experience difficulties in interpersonal relations at school, including peers in their class and teacher support at school. Also, emotional awareness was uniquely related to teacher support. Being aware of their emotions and paying attention to them may enable adolescents to express their emotional experiences, which may then elicit supportive behaviors from teachers (Denham & Burton, 2003). These observations underpin the importance of accepting and awareness of emotional responses to building social relationships at school. This finding adds to prior work that specific emotion regulation strategies are linked to feelings of being accepted, respected, included, and supported by others in the school (Kopelman-Rubin et al., 2020; Zhao & Zhao, 2015).

Moreover, it was found that impulse control difficulties were negatively associated with teacher support. This is consistent with previous work indicating that teachers find it more difficult to deal with students who express anger and that students with externalizing behavior problems are more often rejected by teachers (e.g., Frivold Kostøl & Cameron, 2021; Jerome et al., 2009). Remarkably, impulse control difficulties were positively associated with peer relationships. This indicates that difficulties in engaging in goal-directed behavior when experiencing negative emotions was related to the perception of getting along with more students in the classroom. It is plausible that impulsive behaviors when experiencing negative emotions may elicit reactions from others that could be perceived as validating and supportive of one’s behavior. It is notable that peer relations were measured using a self-report measure of perceived acceptance and not through a peer nomination procedure to map relations among students. It is plausible that a person’s perception of peers with whom they get along does not match reality or the perception by the others in the group. These findings add to previous research showing that in adolescent samples, compared to samples of children, teacher support and peer acceptance may be disconnected. During adolescence, students turn less to their teachers as a source of social information about peers and peer relationships may be more important in guiding their behavior (Engels et al., 2016; Weyns et al., 2018). Together, these findings suggest that teacher support and peer relationships play a differential mediating role in the relationship between impulse control difficulties and components of student engagement.

As expected, network analysis showed that components of student engagement were linked to peer/teacher relations in the classroom. Consistent with previous work (Engels et al., 2016), this study observed a unique relation between teacher support and adolescents’ behavioral and emotional engagement. When adolescents have more supportive relationships with teachers within the school, they also display more positive emotions about participating in learning activities and show more effort, attention, and persistence while initiating and participating in learning activities. This is in line with earlier work (Engels et al., 2021) showing that warm and close teacher-student relationships increase students’ emotional engagement. The findings are also consistent with previous research showing that peer support positively predicts emotional engagement (Yibing et al., 2011). Moreover, peer relationships within the classroom were particularly related to emotional engagement, but not to behavioral engagement. Through its connections with components of student engagement as well as specific emotion regulation difficulties, indirect paths emerged between student engagement and emotion regulation via relationships with peers and teachers. This further underpins the role of peer and teacher relationships in potentially adverse indirect effects of non-acceptance of emotional responses, emotional awareness, and impulse control difficulties on student engagements. Teachers and peers may serve as a source of support to help students to engage in learning activities.

The present findings have implications for daily classroom practices and interventions to promote well-being, social connectedness, and academic performance. First, the findings may inform school-based interventions for promoting students’ emotional well-being and academic performance. Indeed, helping students to regulate diverse emotions may stimulate their learning processes and increase academic performance (Jacobs & Gross, 2014). Broad social and emotional learning (SEL) programs have been developed and seem effective in improving prosocial behavior, school bonding, connectedness, and emotional distress (Durlak et al., 2011; Taylor et al., 2017). However, such programs typically lack specificity in terms of emotion regulation mechanisms targeted. Recently, a school-based emotion regulation intervention has been developed to target emotion regulation skills (Volkaert et al., 2021). While this intervention yielded temporary improvements in depressive symptoms and self-esteem, there is room for improvement in terms of obtaining lasting changes. To obtain lasting effects, student curriculums could integrate parts of emotion regulation interventions (e.g., psychoeducation and exercises; e.g., Nathanson et al., 2016) into the curriculum to facilitate key emotional awareness/clarity, acceptance of emotional responses, impulse control, and emotion regulation strategies during daily learning activities in the classroom. This may help to foster emotional well-being over time.

Second, this study’s observations point to the importance of caring teacher-student relationships to improve student engagement and mitigate emotion regulation difficulties. Teacher support mediated the relation between various emotion regulation difficulties and components of student engagement. To mitigate potential adverse effects of emotion regulation difficulties on student engagement (and vice versa), teachers could develop behaviors that support effective emotion regulation. To target impulse control difficulties, teachers could learn how to help students to control their behavior when they are upset. To facilitate students’ emotional awareness and acceptance of emotions, teachers could engage in conversation and discussion with students about diversity in emotional responses to classroom and life events, provide affirmation of the emotions students may experience and support them to express their thoughts and feelings (e.g., Frivold Kostøl & Cameron, 2021). Such skills of effective supportive behaviors could be trained as part of preservice teacher training. This training needs to inform candidate teachers about the role emotions play in the classroom and provide strategies on how to increase emotional student engagement and support students in regulating their emotions in various classroom situations.

Finally, peer relationships in the classroom also represent a potentially important target to mitigate adverse effects of emotion regulation difficulties on student engagement (and/or vice versa). To improve social integration within the classroom, teachers may need to counter the negative relation between nonacceptance of emotional responses and peer relationships. This could be achieved by monitoring students to better ensure that they do not judge emotions peers express and by teaching students to treat each other with respect. This requires teachers have a proper understanding of social-relational processes within the classroom. Again, such social and emotional skills could be trained as part of preservice and inservice programs.

Several limitations of this study point to future directions. First, the cross-sectional nature of this study weakens claims about causality. It is thus unclear whether emotion regulation difficulties have a negative impact on teacher/peer relationships and student engagement, or vice versa. Future longitudinal research with multiple waves of data collection is required to gain insight into the direction of relationships between emotion regulation difficulties, student engagement, and teacher/peer relationships. This seems particularly interesting now that students are returning to in-person schooling after the acute phase of the SARS-CoV-2 pandemic but are now experiencing higher levels of mental health issues (Holmes et al., 2020). Second, the estimated network models did not consider the multilevel structure of the data (students nested within classes/schools). Because network analysis is still a relatively young analytic technique, there are currently no options to consider the multilevel structure of the data when fitting the network models (Abacioglu et al., 2019). However, this study did not observe systematic differences between the schools with respect to the study variables, suggesting that it is warranted to aggregate across schools. Yet, future research should still aim to replicate the current findings in samples from schools that vary with respect to variables such as SES composition, or local educational indicators. Third, this study relied on self-report measures of the constructs of interest. Such measures represent respondents’ perceived rather than objective levels of emotion regulation difficulties, student engagement, and teacher/peer relationships. Future research could adopt a multi-informant approach including teachers’ assessments and/or a multi-method approach including classroom observations and peer nomination procedures to comprehensively measure the key constructs considered in this study. Finally, this study included schools with lower and higher SES composition of students as determined by Flemish educational indicators. However, given the sample size and the aim of retaining sufficient power for the main analyses, SES was not included as an exploratory node in the network model. Previous research found that socioeconomic risk, which included socioeconomic status, seems to be a risk factor for the family emotional context (e.g., adaptive parent emotion regulation, parenting practices, parent-adolescent relationship quality), which in turn dampens emotion regulation development during adolescence (Herd et al., 2020). Based on these findings, it would be interesting to examine if differences in emotion regulation difficulties exist between students from diverse SES backgrounds and how this is related to student engagement and social support.

## Conclusion

At present, little is known about the specific pathways via which emotion regulation shape students’ engagement in learning activities as well as relations with peers and teachers. This study used advanced statistical modeling of data from secondary school students to map the complex interplay between emotion regulation difficulties, student engagement, and relations with peers and teachers. This study uncovered direct and indirect pathways through which specific emotion regulation may hamper vs. facilitate student learning by impairing student engagement and social relations at school. These findings enable a better understanding of specific mechanisms underlying emotional, social, and cognitive challenges faced by adolescents within school contexts. This may inform the design and focus of interventions targeting adolescents’ mental health and school performance in future work.

## Supplementary Information

Supplementary Information
