# Supplementary material for: Emotion Regulation in the Classroom: A Network Approach to Model Relations among Emotion Regulation Difficulties, Engagement to Learn, and Relationships with Peers and Teachers
Source: J Youth Adolesc. 2022 Sep 30;52(2):273–86. doi: 10.1007/s10964-022-01678-2 (PMC9524346; doi:10.1007/s10964-022-01678-2)
Supplement: Supplementary file 1 — Supplementary Information [file 10964_2022_1678_MOESM1_ESM.docx]

# Supplement 1

*Figure 1*. Non-parametric bootstrapped 95% confidence intervals (CIs) for the estimated edge weights.


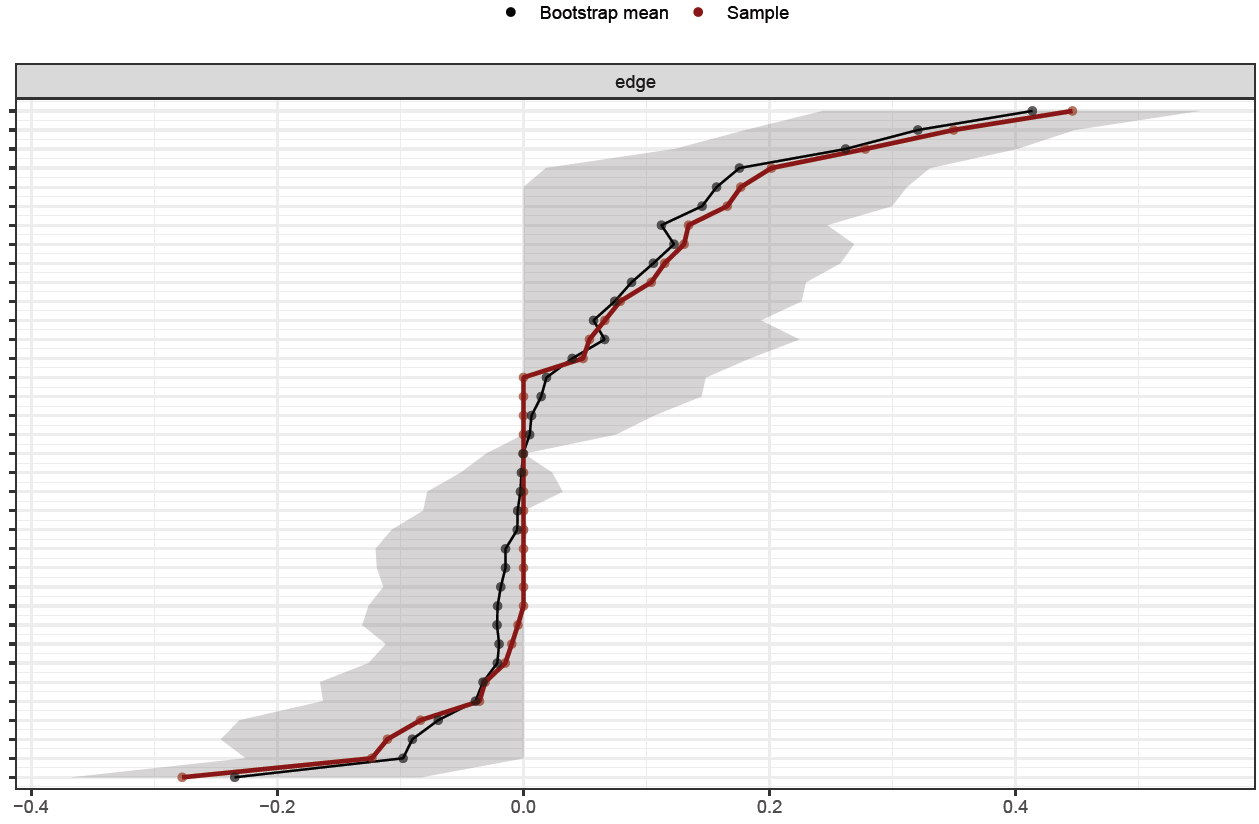


*Figure 2*. Non-parametric bootstrapped difference tests for edge weights.


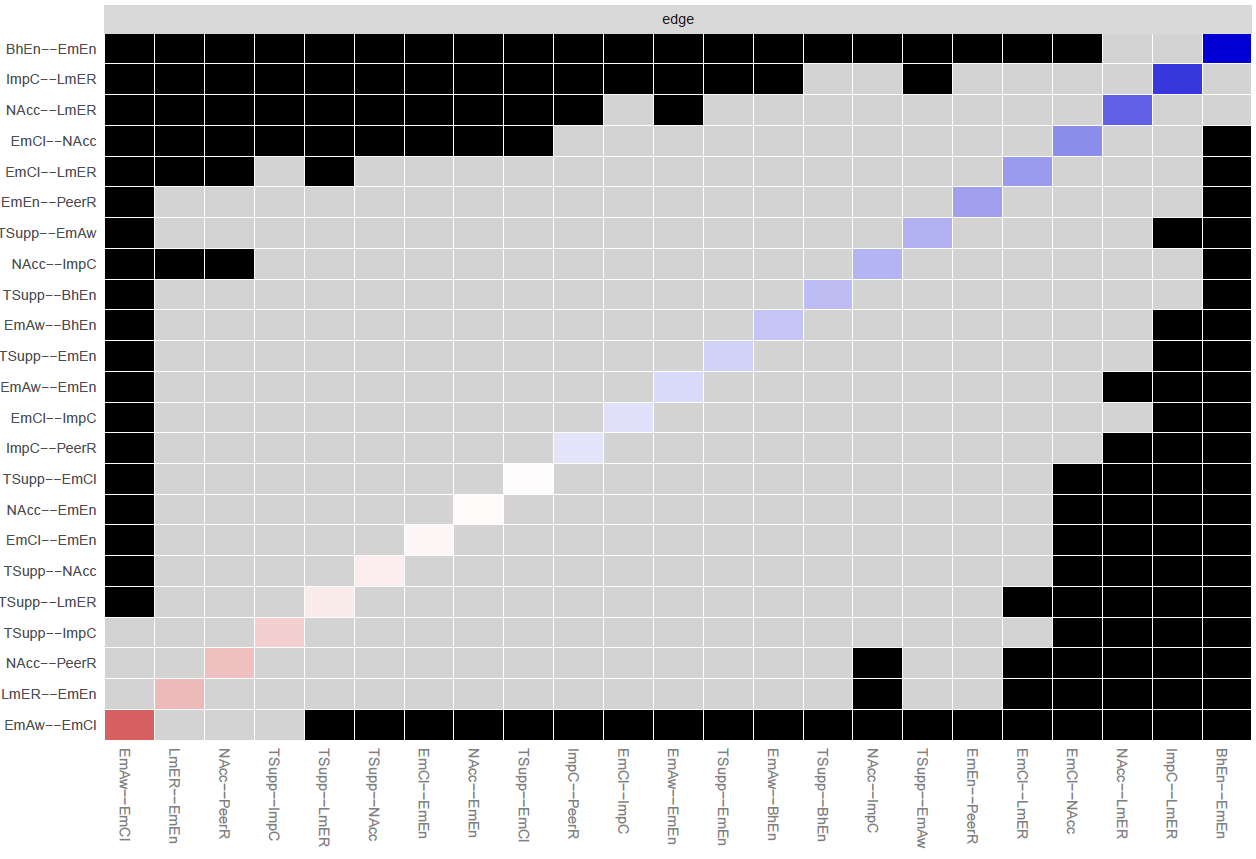


*Figure 3*. Non-parametric bootstrapped centrality difference tests.
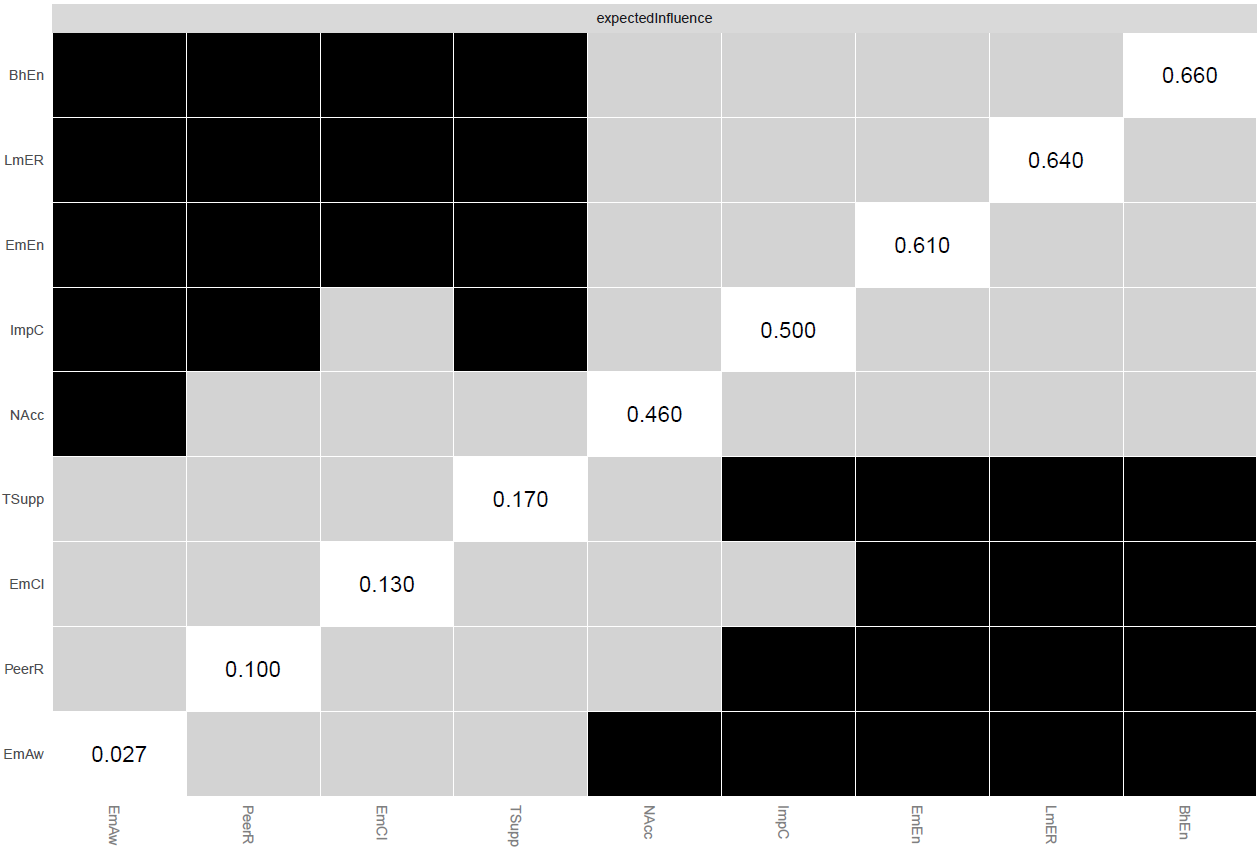


*Figure 4*. Person-dropping bootstrapped estimates of expected influence.


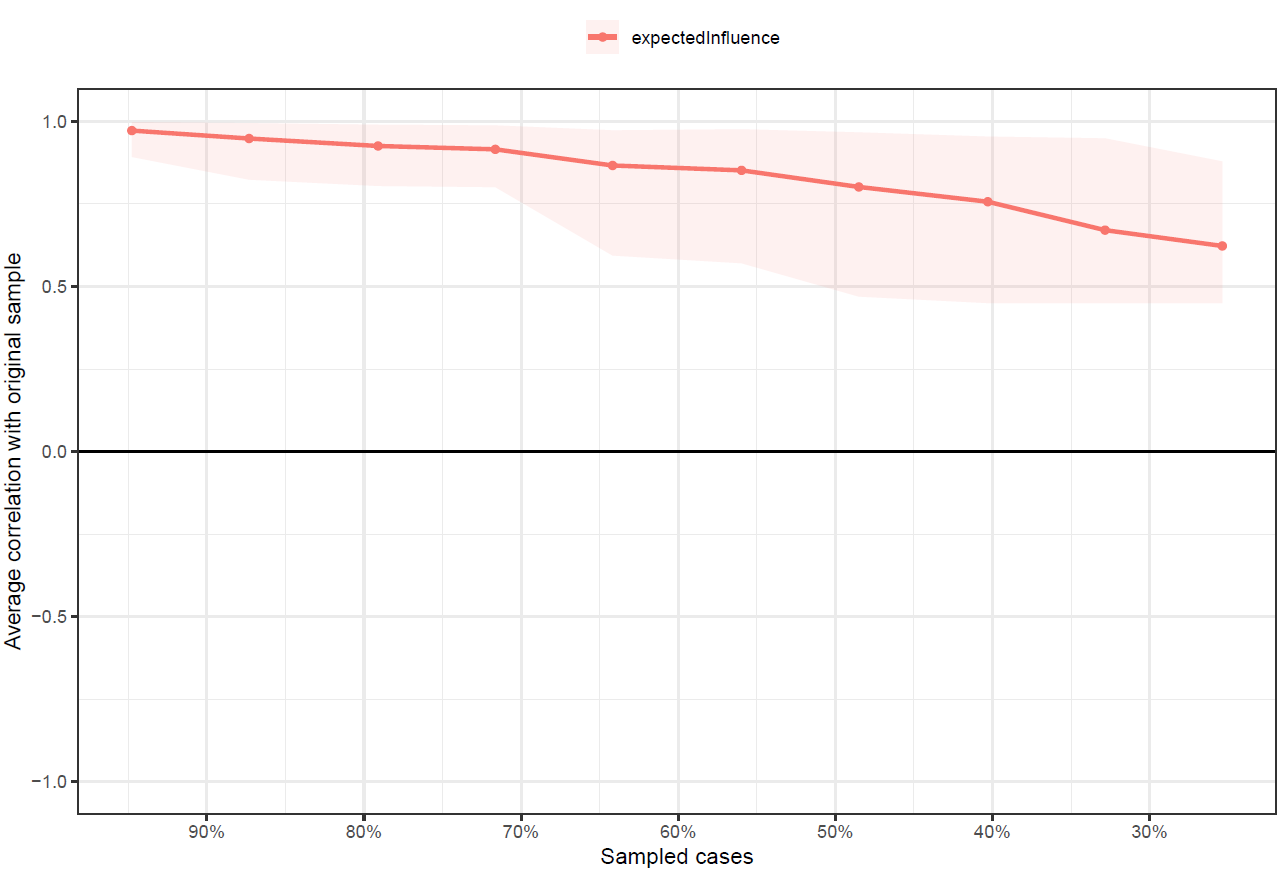


# Supplement 2

*Figure 1*. Patterns of conditional relations convey information about causal orientations.


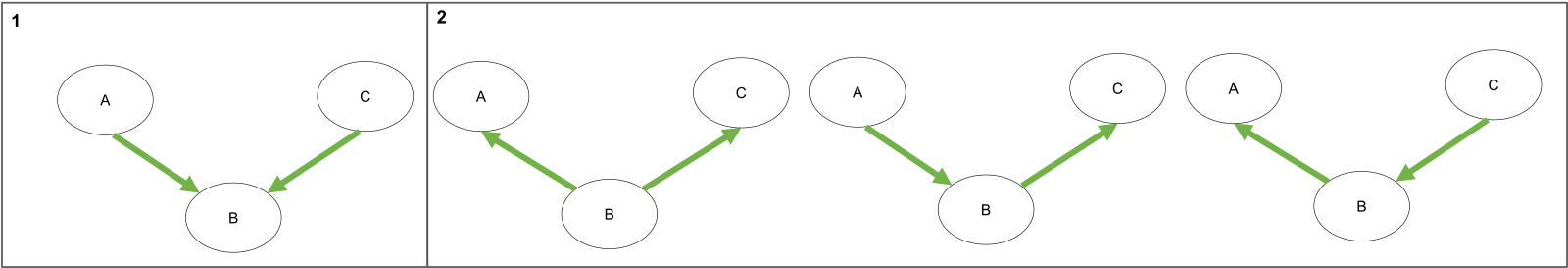


*Notes*. The absence of an arrow denotes the absence of a causal relation. Green arrows denote causal relations between variables (see Table 1). Panel 1: A “collider” graph (A and C directly cause B, no edge between A and C). A is unconditionally independent of C, and A is dependent on C conditional on B. Panel 2: However, in all other possible relations between A, B, and C (where no edge is present between A and C), a different pattern of conditional relations emerges: A is unconditionally dependent on C, and A is independent of C conditional on B. Given the differential pattern of conditional relations between the graphs in Panel 1 and Panel 2, examining conditional relations can support inference about whether a collider or some other causal process generated the observed data. Greedy Fast Causal Inference uses cases like that illustrated above to determine the direction of causal edges and to rule in/out latent confounds of the relations between variables.

Table 1. *Edge Types in a Partial Ancestral Graph Convey Information about Potential Causal Relations.*

| **Edge Type** | **Information Conveyed** |
| --- | --- |
| 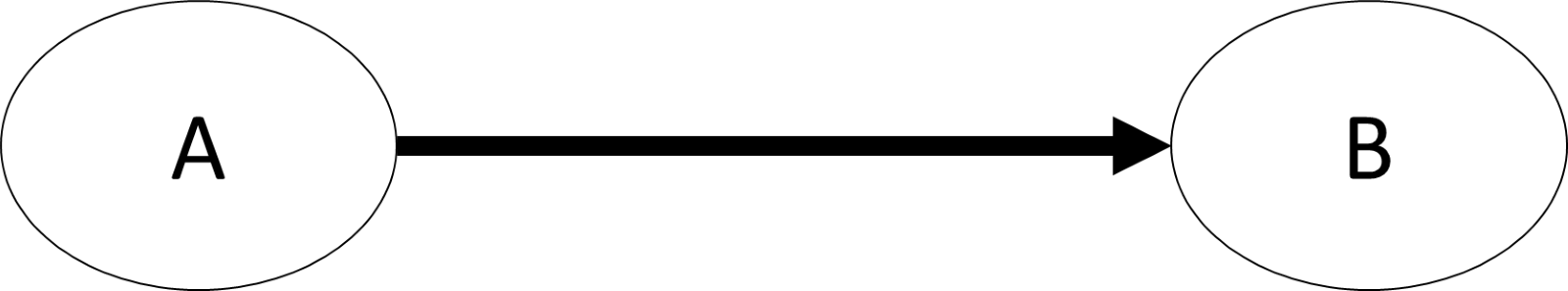 | A is a direct or indirect cause of B. A and B are potentially confounded. B is not a cause of A. |
| 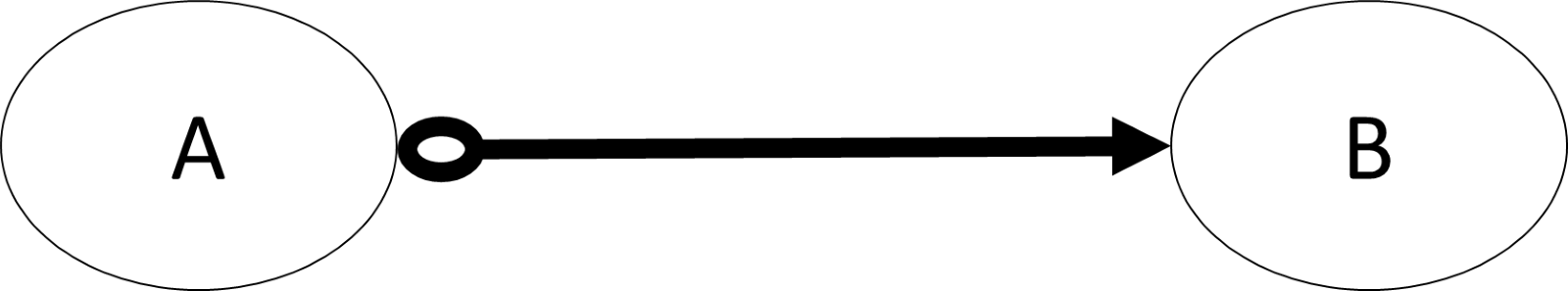 | Either A is a cause of B or there is an unmeasured confounder of A and B, or both.  B is not a cause of A. |
| 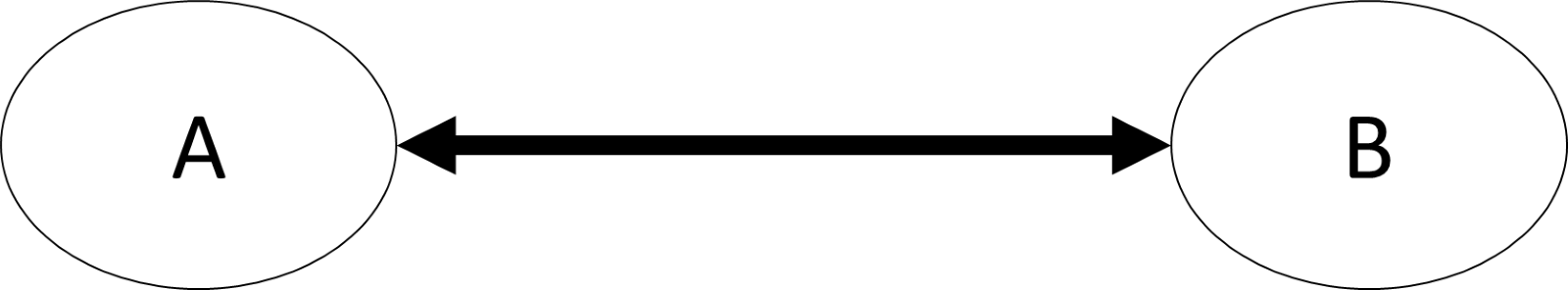 | There is an unmeasured confounder (L) of A and B. There may be measured variables along the causal pathway from L to A or B. |
| 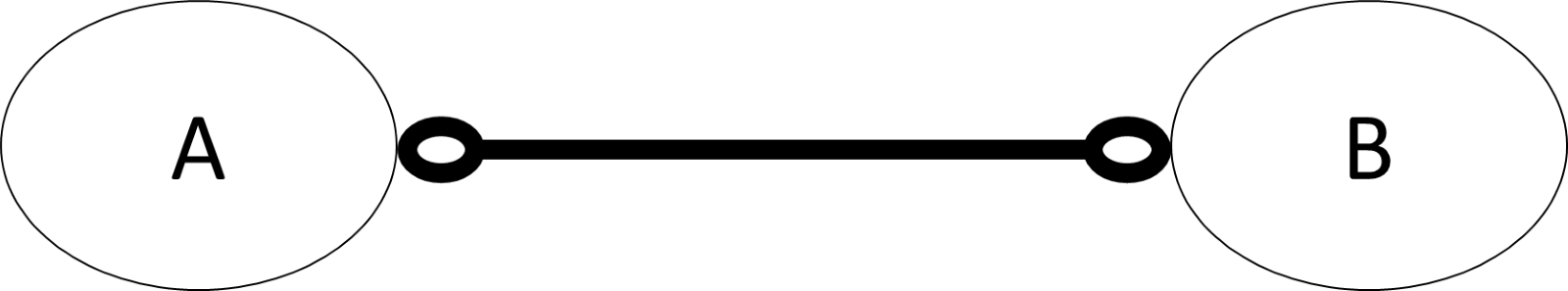 | Exactly one of the following holds:   1. A is a cause of B 2. B is a cause of A 3. There is an unmeasured confounder of A and B 4. Both 1 and 3 5. Both 2 and 3 |

**Note.** In addition to the above, if an edge is **bold** (thickened), then the relation is definitely direct. Else, it is possibly indirect. If an edge is green, there is no latent confounder of the relation; if it is blue, there may be a latent confounder.

Table 2. *GFCI Ensemble Voting Shares*

| Var1 | Var2 | 0-> | <-0 | 0-0 | 🡨pdpl | 🡨pdnl | 🡨ddnl | 🡨ddpl | 🡨🡪 | 🡪pdpl | 🡪pdnl | 🡪ddnl | 🡪ddpl | none |
| --- | --- | --- | --- | --- | --- | --- | --- | --- | --- | --- | --- | --- | --- | --- |
| EmEn | PeerR | -- | **.92** | .06 | .01 | -- | -- | -- | -- | -- | **--** | -- | -- | -- |
| BhEn | EmEn | -- | .04 | .03 | -- | -- | **.93** | -- | -- | -- | -- | **--** | -- | -- |
| EmEn | TSupp | -- | **--** | -- | .02 | -- | .06 | **.85** | -- | -- | -- | -- | -- | .07 |
| EmEn | LmER | -- | -- | .04 | .02 | -- | -- | -- | -- | -- | -- | **.71** | -- | .21 |
| ImpC | LmER | -- | -- | .20 | **--** | -- | **.79** | -- | -- | -- | -- | -- | -- | -- |
| LmER | NAcc | -- | -- | .19 | -- | -- | .05 | -- | -- | -- | .03 | **.70** | -- | -- |
| EmCl | NAcc | -- | -- | .15 | -- | -- | **.45** | -- | -- | -- | -- | .06 | -- | .31 |
| EmAw | EmCl | -- | -- | .25 | .01 | -- | **.63** | -- | -- | -- | -- | .10 | -- | -- |

*Notes*. Var=variable. Pd=possibly direct. Dd=definitely direct. Pl=possibly latent. Nl=no latent. PeerR=Peer relations. EmEn=Emotional Engagement. BhEn=Behavioral Engagement. TSupp=Teacher Support. LmER=Limited Access to Emotion Regulation Strategies. ImpC=Impulse Control Difficulties. NAcc=Non-acceptance of emotional responses. EmCl=Lack of Emotional Clarity. EmAw=Emotional Awareness.
